# A Rare Case of Fibrous Hamartoma of Infancy: A Clinicopathological Diagnosis at a Tertiary Hospital, Eastern Nepal

**DOI:** 10.1155/2019/9410415

**Published:** 2019-01-23

**Authors:** G. Lama, P. Upadhyaya, B. Adhikari, M. Adhikari, S. Dhakal

**Affiliations:** Department of Pathology, BPKIHS, Dharan, Nepal

## Abstract

**Background:**

Fibrous hamartoma of infancy is a rare soft tissue lesion of infants and young children with characteristic triphasic morphology.

**Case Description:**

An 18-month-old female child was presented with complaints of swelling over right leg shin since birth. On examination, a lump of size 7x3 cm was identified which was mobile and nontender. Local excision was performed and tissue sent for histopathological examination. On gross examination, a globular, capsulated, firm to hard tissue had cut section revealing solid grey-white to grey-brown lesion with myxoid areas identified. Microscopic examination revealed a poorly circumscribed lesion comprising intersecting trabeculae of fibrous tissue, areas of immature oval and stellate cell within myxoid matrix, and varying amounts of interspersed mature fat cells.

**Conclusion:**

Even though fibrous hamartoma of infancy is a rare benign entity with limited clinical knowledge, proper diagnosis is mandatory as its prognosis is excellent.

## 1. Introduction

**Figure 1:**
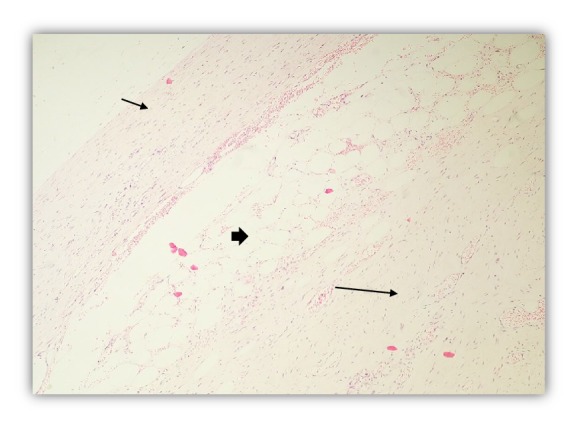
Characteristic trimorphic components: fibrous tissue (short thin arrow), mature adipose tissue (thick arrow), and myxoid matrix (long thin arrow). H & E stain (100X).

Fibrous hamartoma of infancy (FHI) was originally described by Reye in 1956 as “Subdermal fibromatous tumor of infancy.” In 1965, Enzinger suggested the term “fibrous hamartoma of infancy” after experiencing with 30 such cases [1]. It occurs as a solitary mass usually before the age of 2 years and males are affected more. Pathologically, FHI is characterized by the presence of triphasic morphology—fibrous tissue, immature cells in myxoid matrix, and mature adipose tissue [2] (Figure 1). It mainly occurs in axillary and inguinal regions and also in upper arms and trunks. Less common locations include head and neck region, lower extremities, and foot area. It is a painless nodule but some time with rapid growth. The treatment of choice is local excision with very low recurrence rate [3].

## 2. Case Description

An 18-month-old female child was brought to the Surgical Outpatient Department with complaints of swelling in right leg. The mass had been present since birth and was slowly progressing in size.

On physical examination, a lump of size 7x3 cm was identified in the right leg shin. The lump was soft, mobile, and nontender. Rest of the systemic examination was normal.

Ultrasonography examination revealed a heterogenous soft tissue mass along the anterior aspect of proximal right leg, suspicious of fibrosarcoma.

Fine needle aspiration cytology was done from the mass and was diagnosed as spindle cell lesion.

The lump was excised and was sent for histopathological examination.

Gross examination showed a partly skin covered, globular capsulated grey-white to grey-brown, firm to hard tissue measuring 6x4x3 cm. On serial sectioning, cut surface was solid, grey-white to grey-brown along with myxoid areas, and interspersed fibrous tissue forming bands.

**Figure 2:**
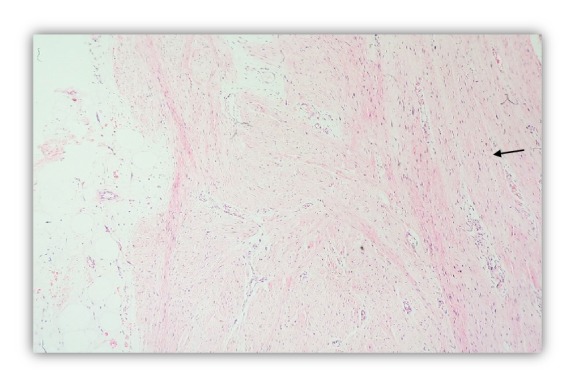
Fibrous Tissue in Fascicles (arrow). H & E stain (100X).

**Figure 3:**
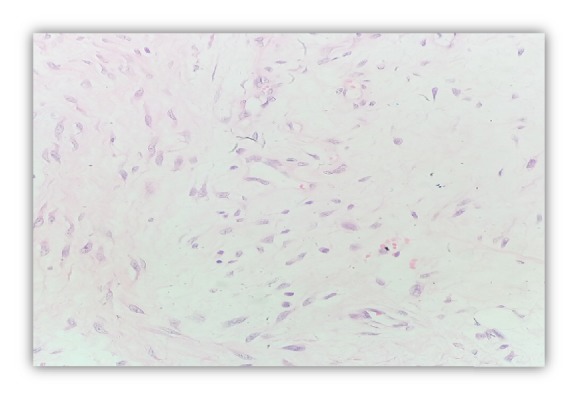
Myxoid Areas. H & E stain (400X).

**Figure 4:**
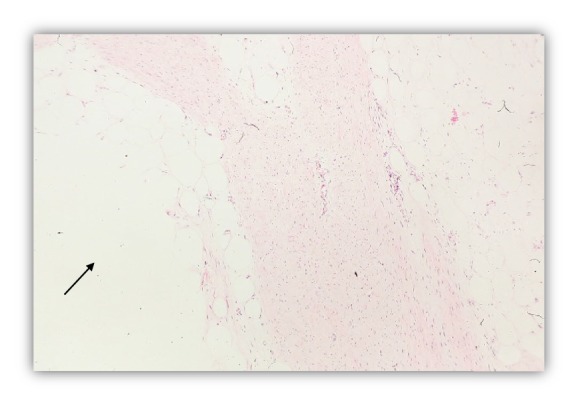
Mature Adipose tissue (arrow). H & E stain (100x).

Microscopic examination revealed a poorly circumscribed lesion comprising three distinct components with vague, irregular, and organoid pattern formed by intersecting trabeculae of fibrous tissue of varying size and shape (Figure 2) along with loosely textured areas consisting of immature small round or stellate cells in a myxoid matrix (Figure 3). Variable amount of interspersed mature adipose tissue was present at the periphery of the lesion (Figure 4).

**Figure 5:**
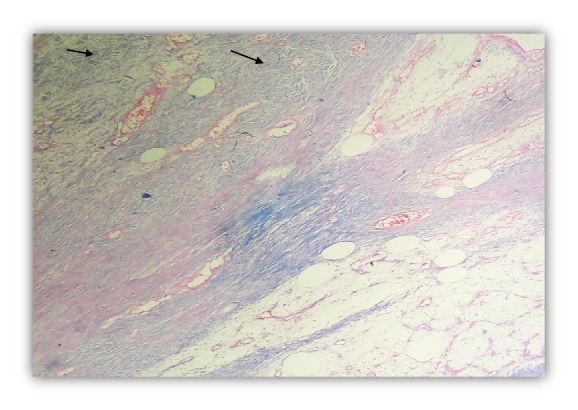
Alcian Blue Stain (arrows). Special stain (100x).

Ancillary test (Immunohistochemistry) was not available. However, special stain showed that mesenchymal matrix was positive for alcian blue stain (Figure 5).

## 3. Discussion

Fibrous hamartoma of infancy is a poorly circumscribed superficial soft tissue mass. It is a rare benign tumor mostly diagnosed before 2 years of age. Twenty percent are congenital. There is slight predilection for boys with male to female ratio of 2.4:1. FHI is most commonly found in axilla, shoulder, upper arm, inguinal region, and chest wall. Less common locations include head and neck, scrotum, legs, and foot [4].

FHI is usually found as a solitary nodule located in the subcutaneous tissue or reticular dermis. The tumor is typically 3 -5 cm but can be up to 15 cm with unclear border. It is firm and may be affixed to underlying tissue, thus causing concerns of potential malignancy. However, local recurrence is uncommon and treatment is largely successful by local excision. FHI are often misdiagnosed as lipoma, neurofibroma, hemangioma, or dermatofibroma [5].

Microscopically, the lesion contains three characteristic components: fibrous tissue adipose tissue and immature or primitive mesenchyme, in varying proportions. The fibrous tissue is usually arranged in interweaving bundles or in an irregular, broken network of branches extending into the adipose tissue. The fibrous fascicles are often mature and fibrotic but can be more cellular and fibroblastic resembling fibromatosis. The immature mesenchyme is arranged in small nests, whorls, or bands of myxoid stroma while mature adipose tissues are interspersed between the other two structural elements [6]. Ancillary test like immunohistochemistry may be useful for diagnosis as fibrous connective tissue is positive for SMA and actin, mature fat tissue is positive for S-100 protein, and undifferentiated mesenchymal tissue is positive for CD 34 and partially positive for actin and SMA. Special stains are unnecessary for diagnosis. However, the matrix within the primitive mesenchymal area is positive for alcian blue [3].

The differential diagnosis for subcutaneous swelling in an infant includes both benign and malignant soft tissue tumors such as epidermoid cyst, recurring digital fibrous tumor, juvenile aponeurotic fibroma, juvenile hyaline fibromatosis, histiocytoma, dermatofibroma, leiomyosarcoma, and fibrosarcoma [7].

## 4. Conclusion

FHI lesions have common histological features, but misdiagnosis is possible because clinical knowledge of FHI is limited and its incidence is rare. The clinical course is typically benign and prognosis is excellent.
